# Investigating Toxin Diversity and Abundance in Snake Venom Proteomes

**DOI:** 10.3389/fphar.2021.768015

**Published:** 2022-01-14

**Authors:** Theo Tasoulis, Tara L. Pukala, Geoffrey K. Isbister

**Affiliations:** ^1^ Clinical Toxicology Research Group, University of Newcastle, Callaghan, NSW, Australia; ^2^ Department of Chemistry, University of Adelaide, Adelaide, SA, Australia

**Keywords:** snake, venom, proteomics, toxin, protein family classification, transcriptomics, mass spectrometry

## Abstract

Understanding snake venom proteomes is becoming increasingly important to understand snake venom biology, evolution and especially clinical effects of venoms and approaches to antivenom development. To explore the current state of snake venom proteomics and transcriptomics we investigated venom proteomic methods, associations between methodological and biological variability and the diversity and abundance of protein families. We reviewed available studies on snake venom proteomes from September 2017 to April 2021. This included 81 studies characterising venom proteomes of 79 snake species, providing data on relative toxin abundance for 70 species and toxin diversity (number of different toxins) for 37 species. Methodologies utilised in these studies were summarised and compared. Several comparative studies showed that preliminary decomplexation of crude venom by chromatography leads to increased protein identification, as does the use of transcriptomics. Combining different methodological strategies in venomic approaches appears to maximize proteome coverage. 48% of studies used the RP-HPLC →1D SDS-PAGE →*in-gel* trypsin digestion → ESI -LC-MS/MS pathway. Protein quantification by MS1-based spectral intensity was used twice as commonly as MS2-based spectral counting (33–15 studies). Total toxin diversity was 25–225 toxins/species, with a median of 48. The relative mean abundance of the four dominant protein families was for elapids; 3FTx–52%, PLA_2_–27%, SVMP–2.8%, and SVSP–0.1%, and for vipers: 3FTx–0.5%, PLA_2_–24%, SVMP–27%, and SVSP–12%. Viper venoms were compositionally more complex than elapid venoms in terms of number of protein families making up most of the venom, in contrast, elapid venoms were made up of fewer, but more toxin diverse, protein families. No relationship was observed between relative toxin diversity and abundance. For equivalent comparisons to be made between studies, there is a need to clarify the differences between methodological approaches and for acceptance of a standardised protein classification, nomenclature and reporting procedure. Correctly measuring and comparing toxin diversity and abundance is essential for understanding biological, clinical and evolutionary implications of snake venom composition.

## 1 Introduction

Venom proteomes of snakes have been published with increasing frequency since 2004 (Juárez et al., 2004), with a review published in late 2017 of 132 snake species characterised up to that point (Tasoulis and Isbister, 2017). Individual snake venoms consist of three to approximately 20 different recognised toxin protein families (Sanz et al., 2019; Calvette et al., 2021; Sunagar et al., 2021), made up from a total pool of 57 protein families so far reported in published snake venom proteomes. This total includes a number of regulatory proteins and low abundance protein families of unknown functional or biological significance. Each of these toxin protein families are believed to be monophyletic, meaning that each family is the result of a single recruitment event into the venom proteome, followed by up-regulation of expression and orthologous diversification (Casewell, 2012; Casewell et al., 2020; Giorgianni et al., 2020). When averaged across all snake species, the majority of snake venoms are composed of four dominant protein families; phospholipase A_2_ (PLA_2_), three-finger toxins (3FTx), snake venom serine protease (SVSP) and snake venom metalloprotease (SVMP) (Tasoulis and Isbister, 2017). A further six secondary protein families make up most of the remaining composition of snake venoms and include; cysteine-rich secretory protein (CRiSP), kuntiz peptides (KUN), l-amino acid oxidase (LAO), natriuretic peptides (NP), C-type lectins (CTL), and disintegrins (DIS) (Tasoulis and Isbister, 2017). Some of these protein families may contain up to 80 different toxins/proteoforms in a single snake species (Tan et al., 2017; Giorgianni et al., 2020; Wang et al., 2020; Sunagar et al., 2021). This amazing diversity of toxins present in some protein families, combined with their synteny and tandemly arrayed location on the genes, is most likely a result of multiple gene duplication events (Wong and Belov, 2012; Hargreaves et al., 2014; Giorgianni et al., 2020).

Characterisation of snake venom proteomes now allows a much more refined way to investigate the evolutionary history of snake venoms. The evolutionary origins of toxins can be approximated by comparisons with phylogeny, providing information on when each of the toxin protein families were ancestrally recruited into the venom proteome. The evolutionary history (orthologous divergence and functional diversification), of these toxin families can then be traced through selected lineages. Venom composition data can be overlaid onto phylogenetic cladograms to reveal if venom composition among related species/genera co-varies with phylogenetic distance. If examples are found in which venom composition diverges from phylogeny, this alerts us to possible unusual evolutionary events that may have occurred and caused changes to venom phenotypes. Comparative venom composition data is also useful for investigating aspects of molecular biology such as gene regulation, gene loss and gene duplication. Used in conjunction with genomic and transcriptomic data, proteomics can provide a wealth of information on the processes governing gene expression, alternative splicing, and other molecular mechanisms responsible for phenotypic divergence. Finally, venom composition data is essential for determining the extent of intra-specific variation, and the influence of ecology and diet versus gene flow on venom evolution (Rautsaw et al., 2019; Schield et al., 2019).

There are two major parts to characterising snake venom proteomes; peptide and protein identification, followed by quantification of the different peptides/proteins. This gives us an estimate of protein diversity and protein family (or individual toxin) abundance. Several previous studies have aimed to establish methods for accurately separating and quantifying protein families in snake venoms (Calvete, 2011; Calvete, 2014; Eichberg et al., 2015; Calderón-Celis et al., 2017; Ghezellou et al., 2019). Some of these studies have emphasised the importance of de-complexing steps prior to mass spectrometry (MS). This workflow usually incorporates reverse-phase high-performance liquid chromatography (RP-HPLC) and 1D sodium dodecyl sulfate polyacrylamide gel electrophoresis (SDS-PAGE). However, size-exclusion chromatography (SEC), and 2D electrophoresis (2DE), have also been used with favourable results (Eichberg et al., 2015; Choudhury et al., 2017). Recently, researchers have demonstrated that using a single method to estimate snake venom composition can result in incomplete proteomic coverage and that combining methods may be preferred (Choudhury et al., 2017; Chanda and Mukherjee, 2020). Some recent studies have compared different methodological steps and demonstrated a concerning degree of variation in the results (Choudhury et al., 2017; Modahl et al., 2018).

Protein identification is most often achieved by matching MS derived peptide sequences with public databases. These databases contain protein sequences of previously investigated snake species that have been uploaded, usually in a non-systematic manner. This introduces further errors into the process, most importantly that toxins from new snake species may not be present in the database and so will not be detected. This limits the ability of studies to identify new toxins. The greater the difference in sequence homology of a toxin the greater the risk of it not being detected. Although the corollary to this is that toxins with high sequence similarity may not be distinguished and hence diversity will be underrepresented. A major step forward has been the advent of transcriptomics technology. This now means that species-specific protein databases can be constructed, and toxins in a venom proteome can be directly matched with the same species transcriptome. This allows the identification of new toxins and a better understanding of the true diversity of snake venom proteomes.

To explore the current state of snake venom proteomics and transcriptomics we aimed to establish which venom proteomic methods are most commonly used, and systematically compare the advantages and disadvantages of different techniques, including methods of isolating, identifying and quantifying toxins, matching to databases and workflow patterns. We aimed to investigate the association between methodological (experimental) variability and biological (i.e. intra-specific) variability to determine if some apparent intra-specific variation may be the result of different experimental techniques. Finally, we compared the diversity and abundance of the identified protein families.

## 2 Materials and Methods

We conducted a systematic review of all studies on snake venom proteomes published between September 2017 and April 2021. We searched the Ovid database > Medline and PubMed, using the search keywords; “snake”, “snake venoms”, “toxins”, “snake venomics”, “elapid”, “viper”, “colubrid”, “proteomics”, “proteomes” and “snake venom proteome”. We identified 143 studies, but only 41 of these met our inclusion criteria for complete venom proteome coverage. We supplemented this search by screening the contents of the following journals for the same time period: Toxins, Toxicon, Toxicon X, Journal of Proteomics, Journal of Proteome Research, International Journal of Biological Macromolecules, Comparative Biochemistry and Physiology Part D. Genomics and Proteomics, Comparative Biochemistry and Physiology Part C. Toxicology and Pharmacology, Proceedings of the Royal Society B, and Acta Tropica. This yielded a further 40 studies to give a total of 81 studies (Supplementary Material S1).

From the included studies we extracted data on the types and amounts of each toxin, to determine toxin diversity and toxin abundance for each snake species. We defined toxin diversity as the total number of different toxins identified in a snake venom. We defined relative toxin abundance as the proportion (percentage) of the whole venom made up by each toxin protein family.

Next, we extracted information on the types of experimental methods and the workflow pattern for each study. Proteomic methodologies were then classified into five different stages: *Prefractionation*: size-exclusion chromatography (SEC), reverse-phase high performance liquid chromatography (RP-HPLC). *Analytical separation*: One and two dimensional sodium dodecyl sulfate-polyacrylamide gel electrophoresis (1D SDS-PAGE and 2D SDS-PAGE), liquid chromatography-tandem mass spectrometry (LC-MS/MS). *Sample preparation*: in-solution trypsin digestion, in-gel trypsin digestion. *Protein identification*: electrospray ionisation (ESI), liquid chromatography tandem mass spectrometry (LC-MS/MS), matrix-assisted laser desorption ionisation-time of flight mass spectrometry (MALDI-TOF MS). *Data analysis*; MS derived peptide sequences matched to transcriptome, and MS derived peptide sequences matched to a public data base.

## 3 Results

### 3.1 Proteomic Methodology

We identified 81 studies that investigated the composition of snake venoms (Supplementary Table S1), 71 investigated relative toxin abundance (and sometimes diversity), while ten investigated only toxin diversity.

#### 3.1.1 A Comparison of Proteomic Methodologies for Determining Toxin Abundance

**FIGURE 1:**
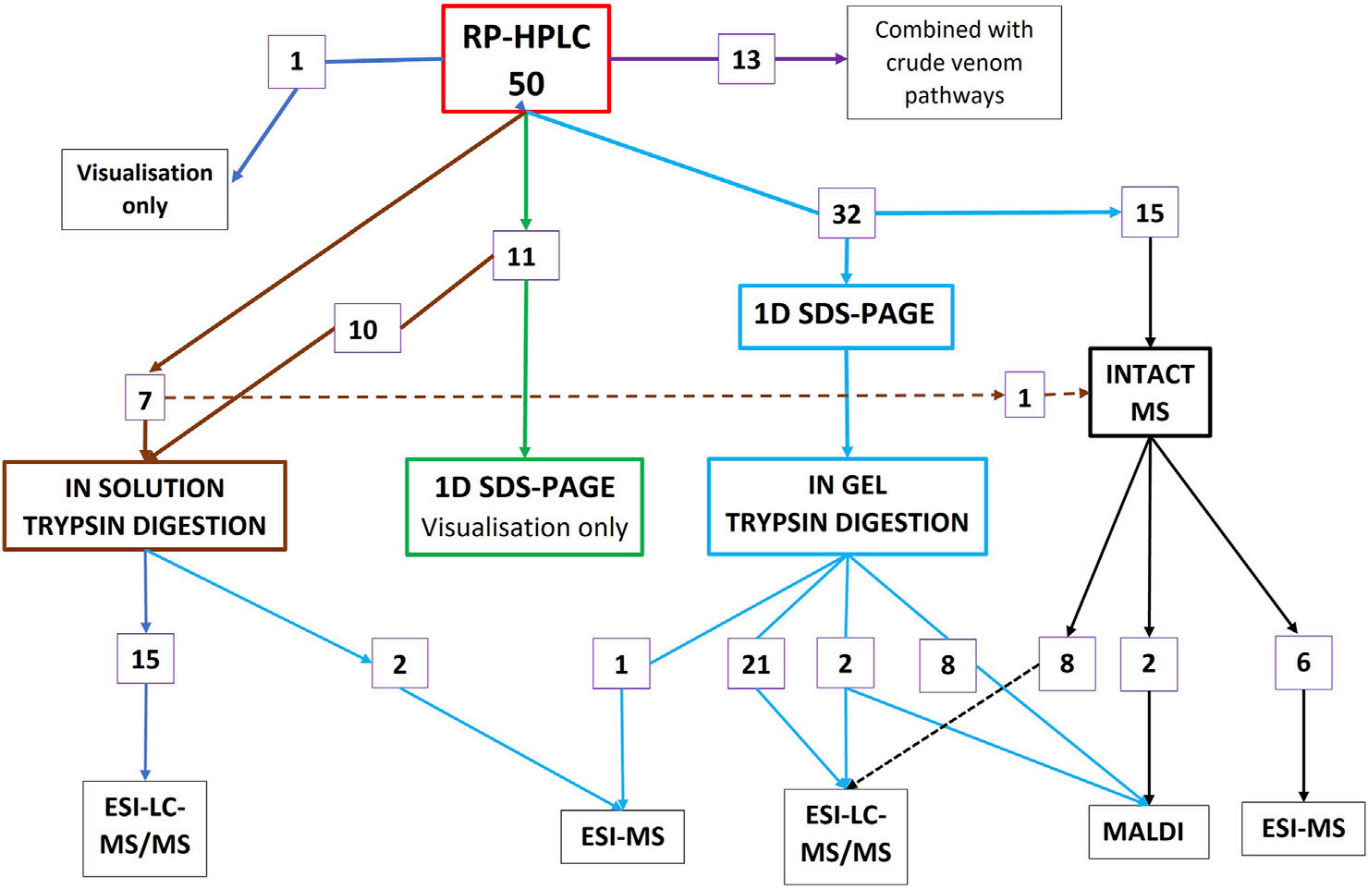
Summary of the workflows for the 50 studies investigating toxin abundance that commenced with the RP-HPLC prefractionation step (13 of these studies additionally used a crude venom pathway, purple). 23 of the 50 studies (46%), followed the 1D SDS-PAGE →*in-gel* trypsin digestion → ESI -LC-MS/MS pathway.

**FIGURE 2:**
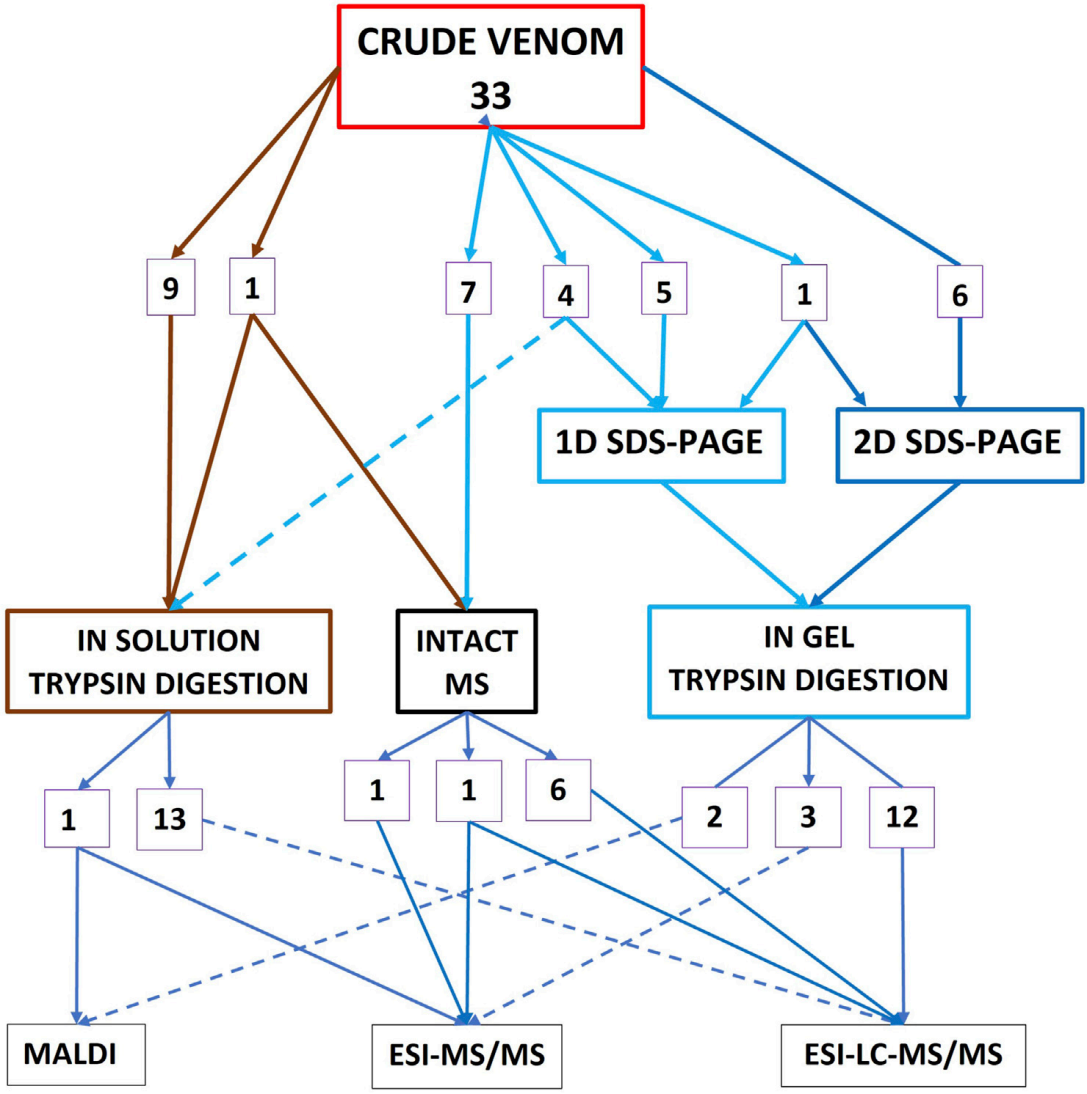
Summary of the workflows for the 33 studies investigating toxin abundance that commenced with crude venom without a prefractionation step (13 of these studies additionally used the RP-HPLC pathway–Figure 1).

**FIGURE 3:**
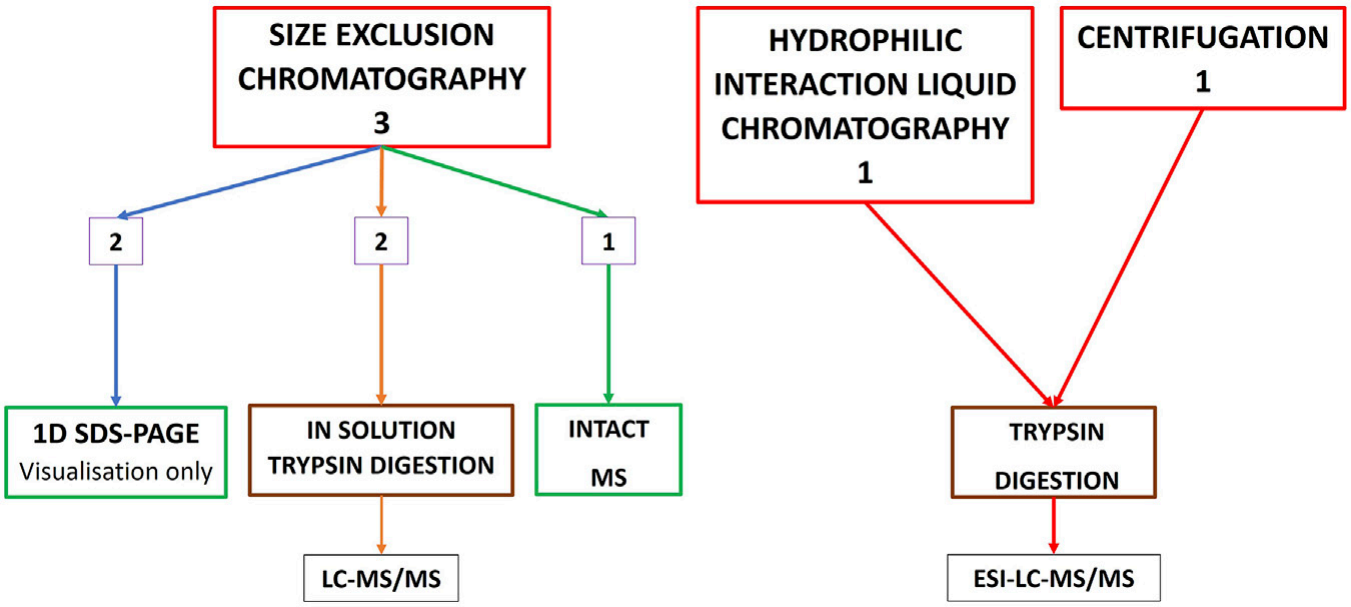
Other methods used for characterising snake venom proteomes; hydrophilic interaction liquid chromatography (HILIC), centrifugation, and a summary of the workflows used for the three studies investigating toxin abundance that commenced with a SEC (also called gel filtration), pathway.

Of the 71 studies investigating toxin abundance, 50 started the workflow with RP-HPLC (Figure 1), 20 commenced the workflow with crude venom without any prior chromatographic prefractionation (Figure 2), 13 studies used both RP-HPLC prefractionation workflow in conjunction with a crude venom workflow, and three studies commenced with size-exclusion chromatography (SEC) alone, as a prefractionation step (Figure 3). Only one study used centrifugation as a pre-fractionation step, and one study used hydrophilic interaction liquid chromatography (HILIC) (Figure 3).

For the second step in the RP-HPLC pathway (Figure 1), 43 of these 50 studies used 1D SDS-PAGE, although 11 of these only used the gels for visualisation of the venom. The remaining 32 followed the classic bottom-up proteomics pathway utilising a workflow of RP-HPLC > 1D SDS-PAGE > *in-gel* tryptic digestion of the peak fractions > LC-MS/MS. 17 studies used RP-HPLC as a prefractionation step for *in-solution* trypsin digestion prior to MS, and 16 of the studies used RP-HPLC as the prefractionation step for intact MS (top-down proteomics, black lines on right Figure 1).

Thirty three studies used workflows that commenced with crude venom without any prior chromatographic prefractionation (Figure 2). However, 13 of these studies additionally used a chromatographic prefractionation workflow in conjunction with a crude venom workflow. 16 of the 33 crude venom studies commenced with either 1D or 2D electrophoresis, followed by trypsin digestion and then MS. Fourteen used a crude venom → in-solution trypsin digestion → MS pathway.

Of the three studies that used SEC pre-fractionation (Figure 3), two continued with in-solution trypsin digestion of the fractions followed by MS, and two studies also used 1D SDS-PAGE as a visualisation tool.

Protein identification was achieved by matching peptide sequences with a public database in 64 studies, with only 17 studies using transcriptomics (24%) (supplementary Material S2).

For the 71 studies which quantified toxin abundance, the most common method was a three-step process of RP-HPLC peak integration/SDS-PAGE densitometry/mass spectrometry, with MS1 spectral intensity being used more than twice as commonly as MS2 spectral counting (35–16 studies respectively–supplementary Material S2).

#### 3.1.2 Increased Protein Identification by Preliminary Decomplexation of Crude Venom

Several studies employed multiple methods on the same snake species to compare the outcomes. Additionally, some snake species were the focus of several independent studies allowing for a comparison of different techniques (Table 1). One study (Choudhury et al., 2017), characterised the venoms of Indian cobra *Naja naja* and common krait *Bungarus caeruleus*, comparing three different methods; tryptic digestion of crude venom, gel-filtration (SEC) fractionation prior to proteomic analysis, and SDS-PAGE fractionation (without prior fractionation using RP-HPLC), followed by LC-MS/MS. For both species, prior fractionation by SEC gave the most comprehensive proteome coverage (Table 1). For *N. naja,* SEC recovered 75 toxins, crude venom digestion 40 toxins, and SDS-PAGE was least efficient, only recovering 25 toxins. For *B. caeruleus*, the results showed a similar trend, SEC–34 toxins, crude venom digestion–30 toxins, and SDS-PAGE 23 toxins (Table 1; Supplementary Table S3). Another study (Ghezellou et al., 2021) compared tryptic digestion of crude venom versus preliminary fractionation by SEC in three populations of saw-scaled vipers *Echis carinatus sochureki* from Iran. For population 1, crude venom digestion recovered 54 proteins compared to 101 proteins using SEC, for population 2, crude venom digestion identified 49 proteins versus 99 proteins using SEC, and for population 3, crude venom digestion identified 53 toxins versus 91 proteins using SEC (Table 1; Supplementary Table S3). A third study (Kunalan et al., 2018), compared trypsin digestion of crude venom with preliminary SEC for the venoms of king cobra *Ophiophagus hannah*, and Malayan pit viper *Calloselasma rhodostoma*. They found *C. rhodostoma* venom contained 47 toxins when using trptic digestion of the crude venom and 99 toxins when venom was prefractionated using SEC. For *O. hannah*, 76 toxins were identified using trypsin digestion of crude venom and 150 toxins by prior fractionation with SEC (Table 1; Supplementary Table S3). These findings of the importance of decomplexation of venom prior to MS support the conclusions of earlier researchers (Calvete, 2011; Eichberg et al., 2015).

**TABLE 1:** A comparison of three different methods of proteome coverage of snake venoms with the number of toxins identified for each method: 1. tryptic digestion of crude venom 2. Prior fractionation of crude venom using SEC, and 3. SDS-PAGE separation of venom mass fractions prior to LC-MS/MS. The studies compared three populations of the saw-scaled viper *Echis carinatus* (Ghezellou et al., 2021), Indian cobra *Naja naja*, common krait *Bungarus caeruleus* (Choudhury et al., 2017), king cobra *Ophiophagus hannah*, and Malayan pit viper *Calloselasma rhodostoma* (Kunalan et al., 2018).

	Tryptic digestion of crude venom	Pre-fractionation by SEC	SDS-PAGE separation of crude venom and LC-MS/MS
*Echis carinatus* Pop 1	54	101	
*Echis carinatus* Pop 2	49	99	
*Echis carinatus* Pop 3	53	91	
*Naja naja*	40	75	25
*Bungarus caeruleus*	30	34	23
*Ophiophagus hannah*	76	150	
*Calloselasma rhodostoma*	47	99	

#### 3.1.3 Increased Protein Identification by Using a Species-Specific Transcriptome Reference and a Public Database Versus a Public Database Only

Two studies (Choudhury et al., 2017; Sunagar et al., 2021), compared the venoms of the common krait *Bungarus caeruleus* and produced different results for venom diversity. The major difference in methodology between the two studies was in protein identification from MS data. One study identified 46 toxins using three different workflow methodologies, but matching MS peptides to the NCBI (public), database, and the other study used venom from a single snake from Maharashtra North-western India and identified 225 toxins by matching MS pepides to a *Bungarus* transcriptome and the NCBI database.

Another study characterised the venom proteomes of two species of colubrids, *Ahaetulla prasina* and *Borikenophis portoricensis*, using species-specific transcriptome references versus a public database only (Modahl et al., 2018). This study found that the number of peptide fragments/spectra mapped was far greater when species-specific transcriptome references were used. Moreover, the pie charts generated for protein family abundance using species-specific transcriptomes were more in agreement with their SDS-PAGE results than the pie charts obtained from using a public database. However, the study also emphasised the importance of combining species-specific transcriptome references with public databases, due to the risk of mRNA degradation or issues with venom gland transcriptome assemblies, resulting in the loss of some toxin transcripts.

#### 3.1.4 Comparison of Top-Down and Bottom-Up Proteomics

“Bottom-up” (BU) proteomics, centred on peptide-based identification from protein digests, has been invaluable for high-throughput identification and quantification of venom proteomes and is demonstrably the most widely adopted approach in venomics (Figures 1, 2). However, identification on the peptide level is suboptimal for characterizing post-translational modifications and sequence variants due to the “peptide-to-protein” inference problem (Nesvizhskii and Aebersold, 2005). This is a particular challenge for venoms in which the same peptide is often present in multiple different proteoforms, leading to ambiguity when determining the identity of toxins, and in turn complicating the determination of the total number and relative abundance of proteoforms present in the venom (Melani et al., 2017; Ghezellou et al., 2019).

First coined in 2013 (Verano-Braga et al., 2013), “TD venomics” has to date principally focussed on mass measurement and sequence analysis of denatured intact proteins, offering the ability to directly identify proteoforms and localize modifications. Despite the advantages of TD strategies, a number of methodological and technical challenges remain, including issues associated with efficient ionisation, fragmentation and isotope resolution of large analytes, which currently limit routine analysis to proteins of approximately 30 kDa (Brown et al., 2020). Therefore, investigation of viperid venom proteomes containing high molecular weight proteins, such as those from SVMPs LAAO and SVSP toxin families, may suffer from reduced TD proteomics detection. Similarly, a recent study of *Echis carinatus sochureki* found that TD proteomics failed to detect some low abundance protein families (NGF and AChE) (Ghezellou et al., 2021). Nevertheless, Calvete and co-workers first reported an integrated analysis of the Indonesian King Cobra venom, with a workflow coupling LC-MS/MS, intact mass measurement of reduced and non-reduced proteins, and BU protein identification with attention to locus-specificity (Ainsworth et al., 2018). Given the complementarity of BU and TD proteomics approaches, multiple recent examples have since demonstrated that, together, they facilitate the most thorough identification of the diversity of snake venom components (Göçmen et al., 2015; Melani et al., 2016; Damm et al., 2018).

Continued developments in intact protein analysis further contribute to venom characterisation. For example, the classical TD venomics approach was extended to elucidate the profile of *Vipera anatolica senliki* venom by an alternative in-source decay (ISD) MALDI based proteomic workflow (Hempel et al., 2020). Although limited somewhat by sensitivity and to sample fractions with minimal complexity, the method was able to overcome challenges with TD identification of high molecular mass venom components. Denaturing fractionation and traditional TD methodology also destroys non-covalent protein-protein and protein-ligand interactions that contribute to biological activity. Therefore employing native MS and native TD proteomics can provide more detailed characterization of protein complexes, resolving combinatorial variations arising from proteolytic protein processing, sequence polymorphism, complexation and post-translational modification (Melani et al., 2016). Finally, beyond identification, a promising workflow combining RP-LC with inductively coupled plasma MS and denaturing MS was also recently reported for absolute quantitation and mass assignment of intact venom proteins (Calderón-Celis et al., 2016).

### 3.2 Venom Composition

#### 3.2.1 Toxin Abundance

**FIGURE 4:**
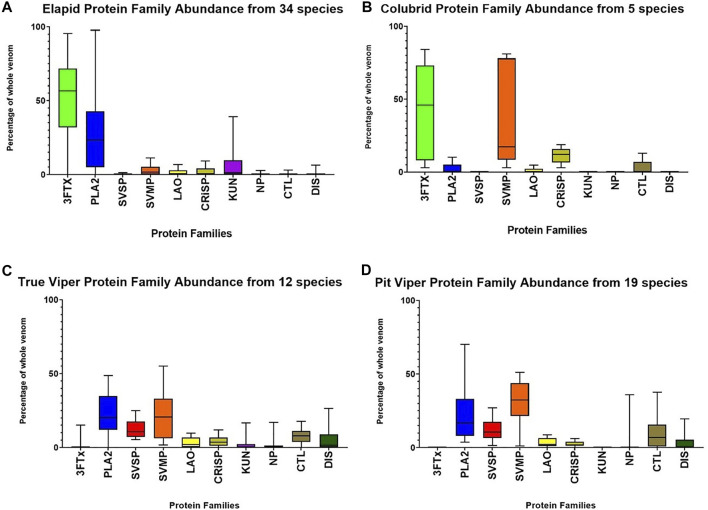
Relative abundance (as % of the whole venom), of the four dominant and six secondary protein families found in the venoms of elapids **(A)**, colubrids–non front-fanged snakes (Non Front-fanged snake) **(B)**, true vipers **(C)**, and pit vipers **(D)**, showing all 70 species for which abundance data was available.

The relative mean (standard deviation) abundance of the four dominant protein families was for elapids; 3FTx -52% (25%), PLA_2_–27% (24%), SVMP–2.8% (3.1%), and SVSP–0.11% (0.30%), and for vipers, 3FTx–0.5% (2.7%), PLA_2_–24% (18%), SVMP -27% (16%), and SVSP–12% (7%) (Figure 4). Elapid venoms are dominated by 3FTx and PLA_2_, and viper venoms are dominated by SVMP, PLA_2_ and SVSP. Major differences in the venom composition between elapids and vipers include SVMP being a large component in the venom of vipers but not elapids, and the reverse being the case for 3FTx. The secondary protein families LAO/CRiSP/NP/CTL/DIS also made up a larger component of viper venoms than elapids, with a combined mean of 24% in vipers versus 10% in elapids. Colubrid, non-front fanged snake (NFFS), venoms were overwhelmingly dominated by SVMP - 38% (37%), and 3FTx - 42% (34%), with the remainder of their venom being made up of CRiSP -11% (5.6%), and small amounts of CTL -2.8% (5.7%), PLA_2_ - 2.% (5%) and LAO -0.9% (2.1%) (Figure 4B) (Supplementary Material S3).

#### 3.2.2 Toxin Diversity

Total toxin diversity within the venoms of each species was readily recoverable from studies of 18 species of elapids, seven species of true vipers and ten species of pit vipers. The median number of different toxins in elapid venoms was 46–[inter quartile range (IQR): 39 to 79, range: 27–225]. For true vipers, the median was 56 (IQR: 25 to 80, range: 25–99). For pit vipers, the median was 39 (IQR: 31 to 68, range: 22–97).

**FIGURE 5:**
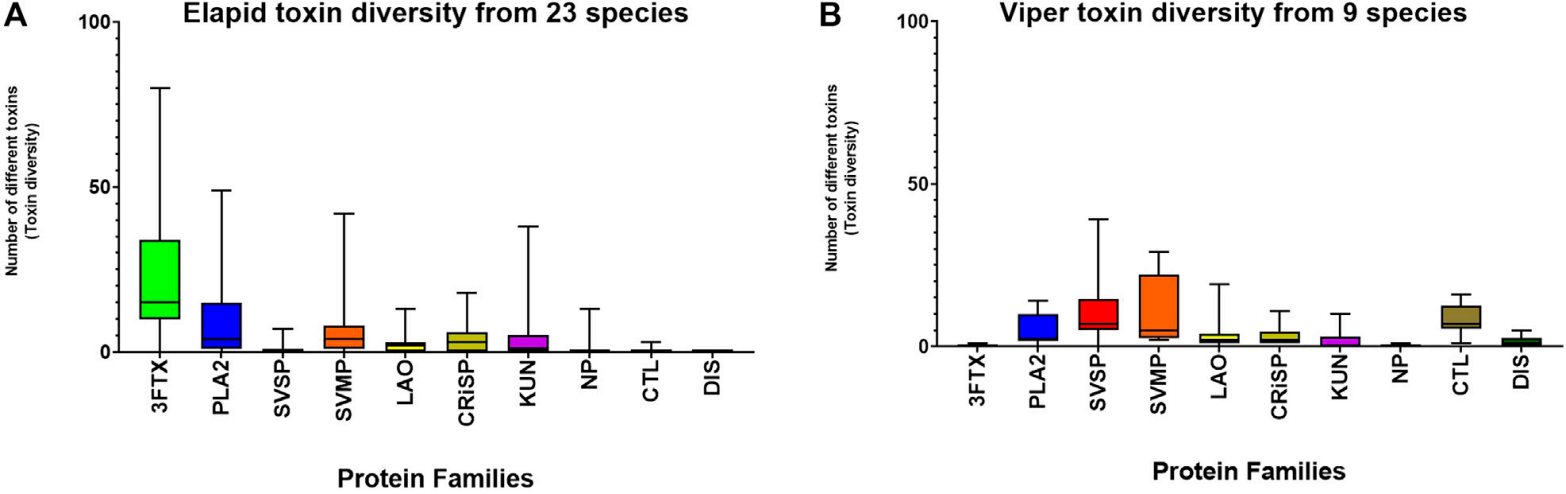
Toxin diversity (number of different toxins), for the four dominant and six secondary protein families found in the venoms of elapids [panel **(A)**], and vipers [panel **(B)**]. Total number of toxins for each protein family in the venom for 23 species of elapids and nine species of vipers are indicated, in some cases averaged from multiple populations/studies of a species.

Toxin diversity within each protein family was readily recoverable for studies of 23 species of elapids, and nine species of vipers (Figure 5). Toxin diversity within protein families for elapids was; 3FTx: median 15, range: 0 to 80. PLA_2_: median 4, range: 0 to 49. SVSP: median 0, range: 0 to 7. SVMP: median 4, range: 0 to 42. For vipers; 3FTx: range: 0 to 1. PLA_2_: median 2, range: 0 to 14. SVSP, median 7, range: 0 to 39. SVMP: median 5, range: 2 to 29.

#### 3.2.3 Relationship Between Toxin Diversity and Protein Family Abundance

**FIGURE 6:**
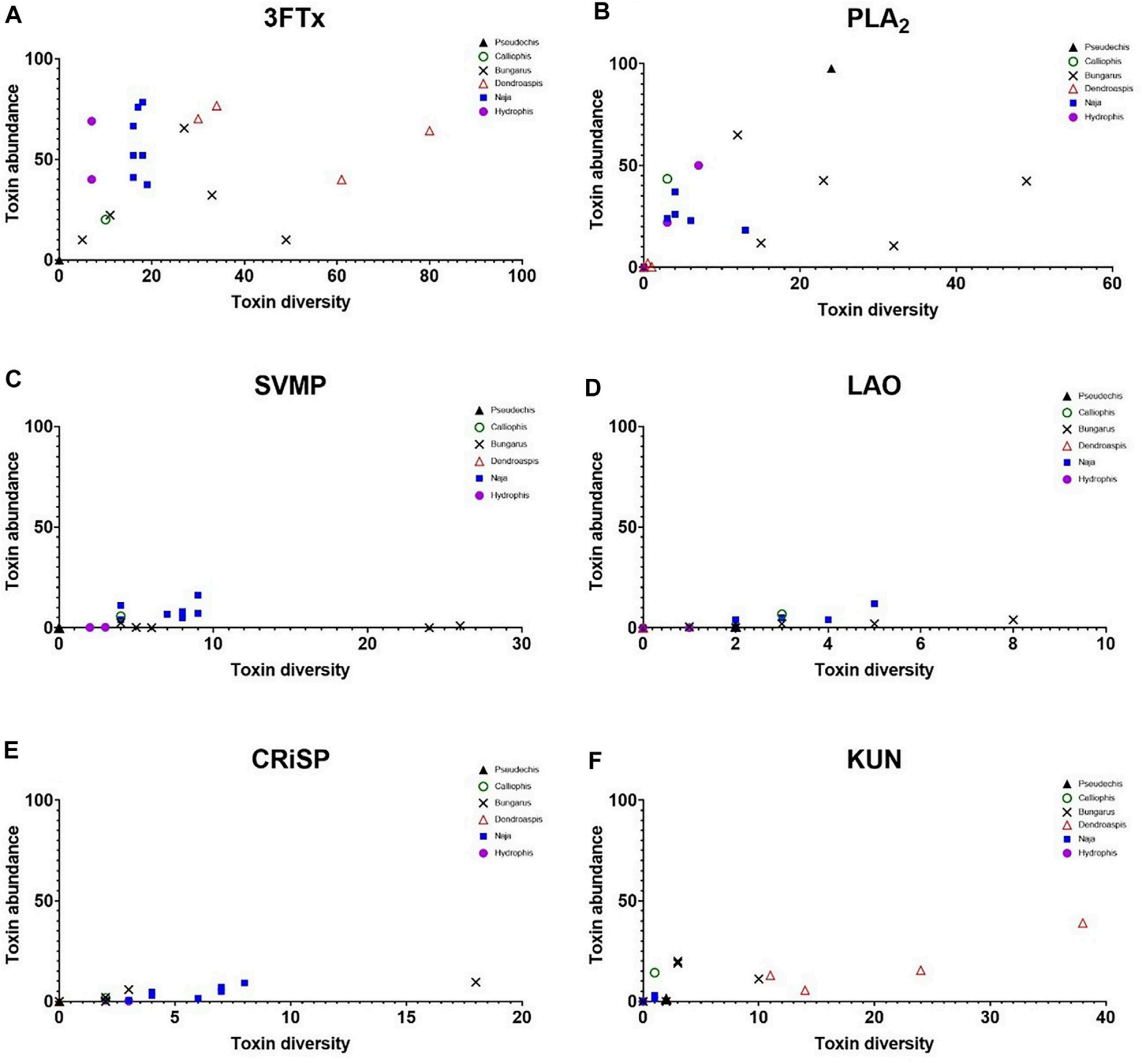
Scatter plots showing the lack of a clear relationship between toxin diversity (*X*-axis), and protein family abundance (*Y*-axis) for the dominant and secondary protein families in elapid venoms. SVSP occurred at such low abundance in elapids it was omitted (see Figure 4A). Colours denote different genera; blue squares = cobras (*Naja* - seven species), red triangles = mambas (*Dendroaspis* - four species), black crosses = kraits (*Bungarus* - five species), purple circles = sea snakes (*Hydrophis* - two species), black triangle = Collett’s snake *Pseudechis colletti*, and green open circle = Malayan striped coral snake *Calliophis intestinalis*.

In elapids, there was no correlation between increasing toxin diversity and higher protein family abundance (Figure 6). This was particularly the case for the dominant protein families (Figures 6A–C). The secondary protein families in elapid venoms showed indications of a linear relationship but it was inconsistent. The strongest correlation was for LAO and CRiSP in cobras and kraits (Figures 6D,E), and KUN in mambas (Figure 6F). However, there were, many examples of phylogenetic groupings in the datasets, with species from the same genera forming clusters e.g. mambas for 3FTx, PLA_2_, KUN, cobras for 3FTx, PLA_2_, SVMP, sea snakes for 3FTx and PLA_2_.

**FIGURE 7:**
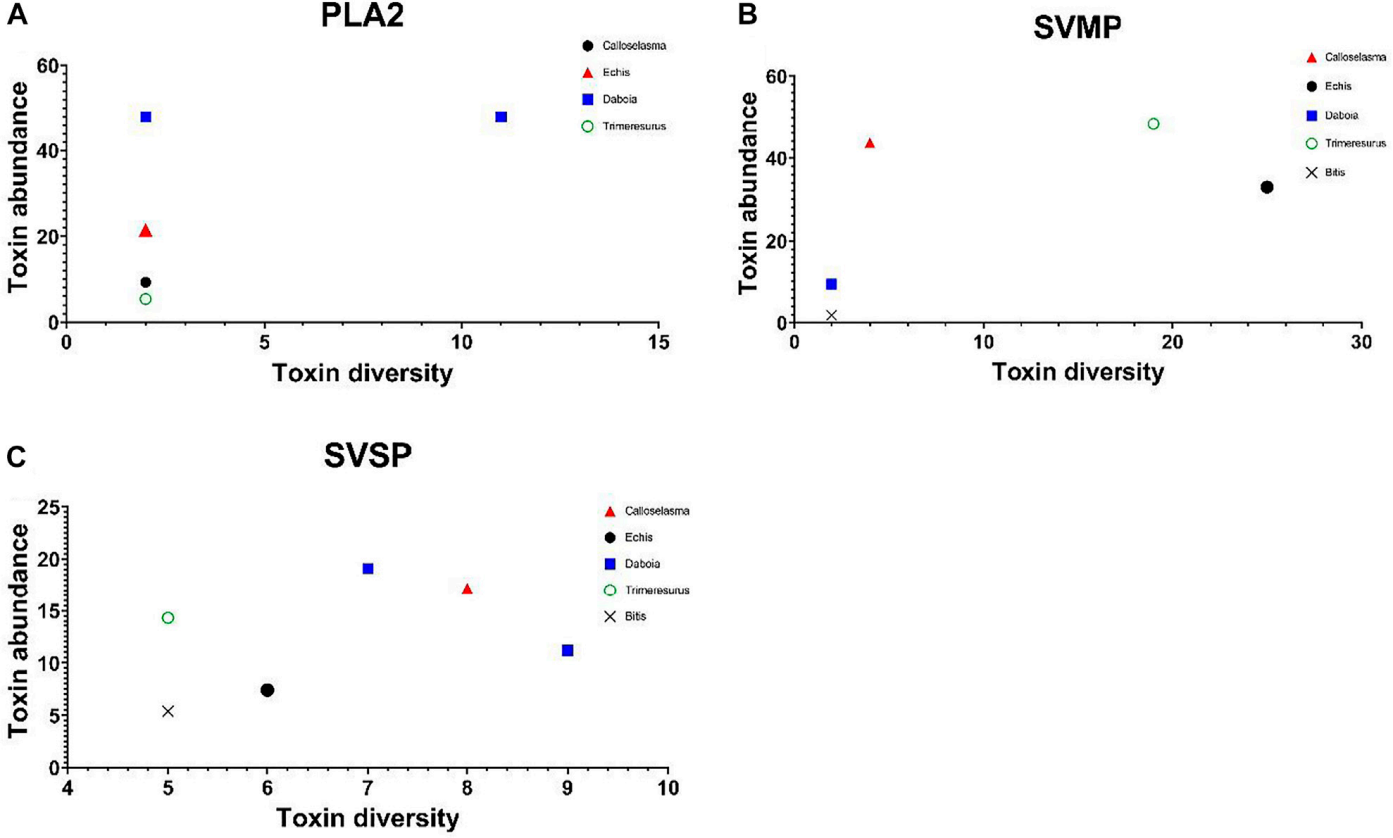
Scatter plots showing the lack of clear relationship between toxin diversity (*X*-axis), and protein family abundance (*Y*-axis) for the three dominant protein families in viper venoms (PLA2, SVMP, and SVSP); These are the seven species from Figure 5B green open circle = *Trimeresurus nebularis*, blue squares = Russell’s vipers *Daboia russelii and D. siamensis*, black circle = saw-scaled viper *Echis carinatus*, black cross = puff adder *Bitis arietans*, and red triangle = Malayan pit viper *Calloselasma rhodostoma*, black diamond = *Protobothrops flavoviridis.*

A comparison of toxin diversity versus protein family abundance in vipers similarly showed no correlation in the dominant protein families (Figure 7).

## 4 Discussion

We compared different methodologies used in 67 studies investigating snake venom proteomics and found that the most common workflow used in just over half of studies to determine protein abundance was prefractionation by RP-HPLC, followed by 1D SDS-PAGE, *in-gel* trypsin digestion and MS (Figure 1). Our analysis showed that preliminary decomplexation of crude venom by chromatography leads to increased protein identification (Choudhury et al., 2017; Kunalan et al., 2018; Ghezellou et al., 2021). Utilizing different methodological strategies such as combining bottom-up and top-down proteomics, and integrating venom gland transcriptomics and public databases, will maximize proteome coverage (Petras et al., 2019; Ghezellou et al., 2021). Limited work has been carried out on individual versus pooled venom. Pooled venom provides a typical representation of the quantitative toxin abundance, but may give an inflated picture of individual venom complexity (Sanz et al., 2020).

With continued technological advancement, TD proteomics is expected to play an increasing role in characterizing the full diversity of venom protein/peptide families, particularly with regard to more detailed description of post-translation modifications and protein complexes (Melani et al., 2017). Furthermore, it is clear from this survey of recent publications, quantitative profiling of venom proteins is increasingly being described to add further informative value beyond qualitative cataloguing of components. Notably, several variants of label free proteomic quantification approaches are known, and largely operate under the assumption that a linear relationship exists between protein abundance and measured MS-based parameters (either the number of spectral counts per protein or MS1 peak intensities) (Ahrné et al., 2013). However, these approaches still suffer from major drawbacks resulting from differential peptide ionizability, or the problem with missing values (Adams and Collyer, 2015). Such strategies for label-free quantification have been developed using organisms for which comprehensive genomic or transcriptomic databases are available. In these cases, it is assumed that the likelihood of ion selection for MS/MS sequencing is higher for abundant peptides, and that the number of peptide identifications (normalised to protein size, since larger proteins generally give rise to more tryptic peptides), can provide a surrogate measure for the abundance of the parent protein. However, of particular importance in the venomics field, where a comprehensive sequence database is missing, quantification is biased toward successful peptide identifications (Calvette et al., 2021).

In this context, although laborious, Hus et al. (2020), recently demonstrated that the traditional multi-stage venomics protocol for quantitative analysis remains the most accurate method available (Calvete, 2018). These results also highlighted the need for caution in the interpretation of data from such quantitative experiments, particularly for comparisons between different studies where label-free proteomics strategies have been employed.

The proteoform complexity and wide dynamic range of venom proteins means that low abundant protein components can often be excluded from identification on the basis of typical data-dependent acquisition (DDA) modes, whereby the highest abundance precursor ions are preferentially targeted for fragmentation and sequence analysis. In the proteomics field broadly, data-independent acquisition (DIA) strategies have emerged to increase reproducibility and depth of coverage. In brief, for DIA modes, all ions present in a wide mass isolation window are fragmented without selection. In this case, parallel MS/MS sequence ions are in principle generated for all peptide precursors within the mass range of interest. These complex ion patterns therefore have to be deconvoluted to reliably relate the observed fragments to a known peptide sequence which is challenging using a conventional genome-wide species-specific database.

Typically, a project-specific spectral library is first required to be generated from multiple fractionated DDA analyses of the same sample searched against a protein sequence database. Matching of the peptide elution time and fragment ion patterns from DIA data to the spectral library then aids in detection. However, given the limitations already discussed for venomics analysis in producing DDA datasets, particularly in cases where protein sequence databases are incomplete, DIA methods have found limited application in venomics analyses.

Elapid venoms consisted of predominately two protein families, with 3FTx making up about half of the venoms on average and PLA_2_ making up about one quarter. However, the relative abundance of PLA_2_ in elapids could be either higher or lower, as many elapid species exhibit a 3FTx/PLA_2_ dichotomy, with one family making up most of the venom (Lomonte et al., 2016). In contrast to elapids, viper venoms were predominately made up of three protein families, with SVMP and PLA_2_ making up a quarter each and SVSP an eighth. Venoms of true and pit vipers showed a high degree of similarity, with only minor differences in the abundance of some of the secondary protein families. Among Viperidae, kunitz peptides were only recorded in true vipers, and natriuretic peptides were more abundant in pit vipers.

Interestingly, colubrid (NFFS) venoms showed features of both elapid and viper venoms, sharing a high abundance of 3FTxs with elapids and a high abundance of SVMPs with vipers. Although only a small number of NFFS were investigated, the studies suggest a 3FTx/SVMP dichotomy, with NFFS venoms being either 3FTx dominant or SVMP dominant. NFFS as a group are not generally considered dangerous, with bites to humans usually only presenting painful local inflammation, and rarely systemic effects (Weinstein and Keyler, 2009; Medeiros et al., 2019; Ineich et al., 2020; Castro et al., 2021). The few species that have caused human fatalities appear to all belong to the group containing predominately SVMP [e.g. boomslang (Pla et al., 2017) and African vine snakes (Debono et al., 2020)], but data is still lacking for Asian Nactricines. 3FTx dominant species are yet to be responsible for human fatalities, despite numerous documented bites (e.g. *Boiga*) (Fritts et al., 1994).

Most venom proteomics studies have focused on the abundance of various toxins and toxin families, and diversity is often not reported or investigated. Toxin diversity is likely to provide significant insights into the biology and evolution of snake venoms. Diversity in snake venoms can be considered as either the number of different protein families in a snake venom, or toxin diversity, being the number of toxins within these protein families. A much greater proportion of viper venoms are made up of secondary protein families compared to elapids (mean values 24–10%; Figure 4). In contrast, elapids appear to have greater toxin diversity within the protein families, compared to vipers. Examples of this are snouted cobra *Naja annulifera* in which most of the venom is made up of the single protein family 3FTx (78%), but within this family there are 18 different 3FTxs (Tan et al., 2020), or Eastern green mamba, which has been recorded to express 80 different 3FTx proteoforms (Ainsworth et al., 2018).

We compared toxin abundance and toxin diversity within different families and groups of snakes within each family. It appears that there is no obvious association between toxin diversity and toxin abundance. In fact, venom composition more closely followed phylogenetic groupings (e.g. cobras and mambas–Figure 6), than any simple relationship between abundance and diversity. This highlights the strong influence of phylogeny on venom composition.

For accurate comparisons of toxin diversity to be made between snake species, definitions of toxin nomenclature and toxin classification need to be agreed upon and adopted. Determining whether two toxins are simply proteoforms or different toxins is not simple. These issues have already begun to be addressed and rational solutions offered (King et al., 2008; Smith and Kelleher, 2013). Recommendations include referring to all protein variations arising from a single gene as proteoforms (in preference to the terms isoforms and protein species), with products of different genes (proteins), being identified as toxins. The term proteoforms also includes variations caused by post-translational modifications. Additionally, a rational nomenclature for peptide toxins has been proposed by King et al. (2008). Further refinements would include establishing guidelines for minimum reporting requirements in publications, such as standardised presentation of data. For example, venom proteome pie charts should state the percentage of whole venom for each protein family, followed by the number of toxins for each protein family in brackets. Papers should also include a summary table of the supplementary toxin tables in the results, which many studies have already adopted. One concerning finding of this review was examples of studies in which there was mislabelling of toxin diversity with relative abundance, or presenting diversity results using vague or ambiguous language. Adoption of the terms toxin diversity and toxin abundance will eliminate this confusion and should be provided in all studies.

A further complication when trying to evaluate toxin diversity is that different results can be achieved depending on whether toxins are matched to a public database or a transcriptome. Public databases may not include a full complement of the toxins present in the venom, resulting in an underestimation of true toxin diversity. Conversely, when matching to a transcriptome, the same peptide sequence can match to several RNA splice variants. This can result in an inflated picture of diversity in the venom proteome. Without a reference genome or the latest technology, single-molecule real time (SMRT) RNA sequencing, it may be difficult to determine if these are different toxins arising from different genes or are merely different mRNA isoforms (toxin proteoforms - some of which may not possess toxic properties). However, these issues will not affect abundance determinations, because this reflects the total amount of the toxin family, not the number of different toxins for each family. We found in this review that transcriptome databases are now used in approximately 25% of all studies (17/71 studies). A final issue which may require consideration is investigating the current status of the curation of public toxin databases, to determine if greater oversight required.

Venomics is essentially a tool that allows us to better understand snake venoms from an evolutionary, biological and clinical perspective. Not only does it provide a simple measure of the abundance of different protein families within snake venoms, it provides information on the diversity of these different protein families and the diversity of individual toxins within the protein families. In the development of antivenoms to treat snake envenoming, an understanding of the difference between protein family abundance/diversity and actual toxin diversity is essential. Antibodies are likely to cross-neutralise toxins within the same toxin family, potentially those in the same toxin family but in different snake venoms (Isbister et al., 2010; Silva et al., 2016). In this way, identifying the toxin families that are the most medically important and focusing antivenoms on these will improve the efficacy of antivenom treatment.

There has been an exponential increase in the characterisation of snake venom proteomes in the last 2 decades (Calvette et al., 2021), and a corresponding increase in the use of this data for inter-species and inter-generic comparisons to unravel evolutionary histories/processes, and medical implications (Petras et al., 2011; Lomonte et al., 2016; Strickland et al., 2018; Barua and Mikheyev, 2019; Jackson et al., 2019; Barua and Mikheyev, 2020; Damm et al., 2021). Given these developments, it is essential that equivalent comparisons are being made, so there is an important need for clarification of the best methodologies, as well as an acceptance of accurate definitions, reporting and nomenclature, in this important and rapidly developing field of research.
