# Supplementary material for: Investigating Toxin Diversity and Abundance in Snake Venom Proteomes
Source: Front Pharmacol. 2022 Jan 14;12:768015. doi: 10.3389/fphar.2021.768015 (PMC8795951; doi:10.3389/fphar.2021.768015)
Supplement: Supplementary file 3 [file Table1.DOCX]

| **YEAR** | **AUTHORS** | **TITLE** | **REF** |
| --- | --- | --- | --- |
|  |  | **ELAPIDS** |  |
| 2020 | Wang et.al. | Interrogating the higher order structures of snake venom proteins using an integrated mass spectrometric approach. | (1) |
| 2017 | Neale et.al. | The venom of the Spine-bellied sea snake (Hydrophis curtus): Proteome, Toxin diversity and Intra-specific variation | (2) |
| 2019 | Tan et. al. | Venom proteome of Spine-Bellied Sea Snake (*Hydrophis curtus*) from Penang, Malaysia: Toxicity correlation, immunoprofiling and cross-neutralisation by sea snake antivenom. | (3) |
| 2020 | Wang et.al. | A comparative analysis of the proteomes and biological activities of the venoms from two sea snakes *Hydrophis curtus* and *Hydrophis cyanocinctus*, from Hainan, China. | (4) |
| 2019 | Tan et.al. | Unlocking the secrets of banded coral snake (*Calliophis intestinalis*, Malaysia): A venom with proteome novelty, low toxicity and antigenicity. | (5) |
| 2019 | Patra et.al | Quantitative proteomic analysis of venom from Southern India Common Krait (*Bungarus caeruleus*), and identification of poorly immunogenic toxins by immune profiling against commercial antivenom | (6) |
| 2020 | Hia et. al. | Comparative venom proteomics of banded krait (*Bungarus fasciatus*) from five geographical locales: Correlation of venom lethality, immunoreactivity and antivenom neutralisation | (7) |
| 2018 | Chapeaurouge et. al. | Proteomic deep mining the venom of Red-headed Krait, *Bungarus flaviceps*. | (8) |
| 2019 | Mei Feng Oh et. al. | Venom proteome of *Bungarus sindanus* (Sind Krait) from Pakistan and in vivo cross-neutralisation of toxicity using an Indian polyvalent antivenom. | (9) |
| 2021 | Sunagar et. al. | A Wolf in another Wolf’s clothing: Post-genomic regulation dictates venom profiles of medically important cryptic kraits in India. | (10) |
| 2018 | Ainsworth et. al. | The medical threat of mamba envenoming in sub-Saharan Africa revealed by genus-wide analysis of venom composition, toxicity and antivenomics profiling of available antivenoms. | (11) |
| 2020 | Tan et. al. | Quantitative proteomics of *Naja annulifera* (sub-Saharan snouted cobra) venom and neutralisation activities of two antivenoms in Africa. | (12) |
| 2020 | Chanda et. al. | Quantitative proteomics to reveal the composition of Southern India spectacled cobra (*Naja naja*) venom and its immunological cross-reactivity towards commercial antivenom. | (13) |
| 2019 | Chanda et. al. | Proteomic analysis and antivenomics study of Western India *Naja naja* venom: correlation between venom composition and clinical manifestations of cobra bite in this region | (14) |
| 2018 | Chanda et. al. | Proteomic analysis to compare the venom composition between *Naja naja* and *Naja kaouthia* from the same geographical location of Eastern India: Correlation with pathophysiology of envenomation and immunological cross-reactivity towards commercial polyantivenom | (15) |
| 2018 | Wong et. al. | Elucidating the biogeographical variation of the venom of *Naja naja* (spectacled cobra) from Pakistan through a venom-decomplexing proteomic study. | (16) |
| 2019 | Tan et.al. | Proteomic insights into short neurotoxin-driven, highly neurotoxic venom of Philippine cobra (*Naja philippinensis*) and toxicity correlation of cobra envenomation in Asia. | (17) |
| 2021 | Wong et. al. | A neurotoxic snake venom without phospholipase A_2_: Proteomics and cross-neutralisation of the venom from Senegalese Cobra, *Naja senegalensis* (subgenus: Uraeus). | (18) |
| 2021 | Adamude et.al. | Proteomic analysis of three medically important Nigerian *Naja* (*Naja haje, Naja katiensis* and *Naja nigricollis*) snake venoms. | (19) |
| 2018 | Sanchez et.al. | Proteomic and toxinological characterization of the venom of the South African Rhinghals cobra *Hemachatus haemachatus* | (20) |
| 2019 | Whiteley et. al. | Defining the pathogenic threat of envenoming by South African shield-nosed and coral snakes (genus *Aspidelaps*), and revealing the likely efficacy of available antivenom. | (21) |
| 2020 | Benard-Valle et. al. | Functional, proteomic and transcriptomic characterization of the venom from *Micrurus browni browni*: Identification of the first lethal multimeric neurotoxin in coral snake venom. | (22) |
| 2019 | Sanz et. al. | New insights into the phylogeographic distribution of the 3FTx/PLA_2_ venom dichotomy across genus *Micrurus* in South America. | (23) |
| 2019 | Lippa et. al. | First look into the venom of Roatan Island’s critically endangered coral snake *Micrurus ruatanus*: proteomic characterization, toxicity, immunorecognition and neutralisation by an antivenom. | (24) |
| 2019 | Sanz et. al. | Comparative venomics of Brazilian coral snakes: *Micrurus frontalis*, *Micrurus spixii spixii*, and *Micrurus surinamensis*. | (25) |
| 2020 | Hus et. al. | Different Research Approaches in Unravelling the Venom Proteome of Naja ashei | (26) |
| 2021 | Laxme et. al. | Biogeographical venom variation in the Indian spectacled cobra (*Naja naja*) underscores the pressing need for pan-India efficacious snakebite therapy | (27) |
|  |  | **TRUE VIPERS** |  |
| 2020 | Pla et. al. | Dagestan blunt-nosed viper, *Macrovipera lebetina obtusa* (Dwigubsky, 1832), venom. Venomics, antivenomics, and neutralisation assays of the lethal and toxic venom activities by anti- *Macrovipera lebetina turanica* and anti-*Vipera berus berus* antivenoms. | (28) |
| 2020 | Bhatia, S. and Vasudevan, K. | Comparative proteomics of geographically distinct saw-scaled viper (*Echis carinatus*) venoms from India. | (29) |
| 2020 | Patra, A. and Mukherjee, A.K. | Proteomic analysis of Sri Lanka *Echis carinatus* Venom: Immunological Cross-reactivity and enzyme neutralization potency of Indian polyantivenom. | (30) |
| 2021 | Ghezellou et. al. | Integrating Top-Down and Bottom-Up Mass Spectrometric strategies for proteomic profiling of Iranian Saw-Scaled Viper, *Echis carinatus sochureki*, venom. | (31) |
| 2019 | Ozverel et. al. | Investigating the cytotoxic effects of the venom proteome of two species of the Viperidae family (*Cerastes cerastes* and *Cryptelytrops purpureomaculatus*) from various habitats. | (32) |
| 2019 | Pla et. al. | Phylovenomics of *Daboia russelii* across the Indian subcontinent. Bioactivities and comparative *in vivo* neutralization and *in vitro* third generation antivenomics of antivenoms against venoms from India, Bangladesh and Sri Lanka. | (33) |
| 2018 | Faisal et. al. | Proteomics, functional characterisation and antivenom neutralisation of the venom of Pakistani Russell’s viper (*Daboia russelii*) from the wild. | (34) |
| 2018 | Kalita et. al. | Proteomic analysis and Immuno-profiling of Eastern India Russell’s viper (*Daboia russelii*) venom: Correlation between RVV composition and clinical manifestations post RV bite. | (35) |
| 2018 | Sanz et. al. | Translational venomics: Third generation anti-venomics of anti-Siamese Russell’s viper, *Daboia siamensis*, antivenom manufactured in Taiwan’s CDC’s Vaccine Centre. | (36) |
| 2020 | Hempel et. al. | Extended snake venomics by Top-Down In-Source Decay: Investigating the newly discovered Anatolian Meadow Viper subspecies, Vipera anatolica senliki. | (37) |
| 2020 | Giribaldi et. al. | Venomics of the Asp viper *Vipera aspis aspis* from France. | (38) |
| 2019 | Al-Shekhadat et. al. | *Vipera berus berus* venom from Russia: Venomics, bioactivities and preclinical assessment of Microgen antivenom. | (39) |
| 2018 | Hempel et. al. | Comparative venomics of the *Vipera ammodytes trancaucasiana* and *Vipera ammodytes motandoni* from Turkey provides insights into kinship. | (40) |
| 2019 | Petras et. al. | Intact protein mass spectrometry reveals intraspecies variation in venom composition of a local population of *Vipera kaznokovi* in Northeastern Turkey. | (41) |
| 2020 | Balija et. al. | Biological activities and proteomic profile of the venom of *Vipera ursinii* ssp., a very rare Karst viper from Croatia | (42) |
| 2020 | Lingam et.al. | Proteomics and antivenom immunoprofiling  of Russell’s viper (*Daboia siamensis*) venoms  from Thailand and Indonesia | (43) |
| 2021 | Laxme et.al. | Biogeographic venom variation in Russell’s Viper (Daboia russellii) and the preclinical inefficacy of antivenom therapy in snakebite hotspots | (44) |
| 2018 | Tan et.al. | Venom proteomics and antivenom neutralization for the Chinese eastern Russell’s Viper, *Daboia siamensis* from Guangxi and Taiwan | (45) |
|  |  | **PIT VIPERS** |  |
| 2019 | Tang et. al. | Comparative proteomes, immunoreactivities and neutralisation of procoagulant activities of *Calloselasma rhodostoma* (Malayan Pit Viper) venoms from four regions in Southeast Asia. | (46) |
| 2019 | Jones et. al. | Venom composition in a phenotypically variable Pit Viper (*Trimeresurus insularis*) across the Lesser Sunda archipelago. | (47) |
| 2019 | Tan et. al. | Venomics of *Trimeresurus* (*Popeia*) *nebularis*, the Cameron Highlands Pit viper from Malayasia: Insights into venom proteome, toxicity, and neutralisation of antivenom. | (48) |
| 2021 | Liu et. al. | Snake venom proteome of *Protobothrops mucrosquamatus* in Taiwan: delaying venom induced lethality in a rodent model by inhibition of phospholipase A_2_ activity with varespladib. | (49) |
| 2020 | Mora-Obando et. al. | Venom variation in *Bothrops asper* lineages from North-Western South America | (50) |
| 2020 | Sanz et. al. | Danger in the Canopy. Comparative proteomics and bioactivities of the venoms of the South American Palm Pit Viper *Bothrops bilineatus* subspecies *bilineatus* and *smaragdinus* and antivenomics of *B. b. bilineatus* (Rhondonia) venom against the Brazilian pentabothropic antivenom. | (51) |
| 2018 | Rodrigues et.al. | Proteomic profile, biological activities and antigenic analysis of the venom from *Bothriopsis bilineata smaragdina* (“loro machaco”), a pitviper snake from Peru. | (52) |
| 2020 | Rodrigues et.al. | Proteomic and toxicological characterisation of Peruvian pitviper *Bothrops brazili* (“jergon shushupe”), venom. | (53) |
| 2018 | Da Costa Galizio et. al. | Compositional and functional investigation of individual and pooled venoms from long-term captive and recently wild-caught *Bothrops jararaca* snakes. | (54) |
| 2020 | Pereanez et. al. | Snake venomics, experimental toxic activities and clinical characteristics of human envenomation by *Bothrocophias myersi* (Serpentes: Viperidae) from Colombia. | (55) |
| 2020 | Neri-Castro et. al. | Venomics and biochemical analysis of the black-tailed horned pitviper *Mixcoatlus melanurus*, and characterisation of melanurutoxin, a novel crotoxin homolog. | (56) |
| 2019 | Neri-Castro et. al. | Venom characterisation of the three species of *Ophryacus* and proteomic profiling of *O. sphenophrys* unveils Spenotoxin, a novel Crotoxin-like heterodimeric β-neurotoxin. | (57) |
| 2018 | Quintana-Castillo et. al. | Characterisation of the venom of *C. d. cumanensis* of Colombia: Proteomic analysis and antivenomic study. | (58) |
| 2020 | Tasima et. al. | Comparative proteomic profiling and functional characterisation of venom pooled from captive *Crotalus durissus terrificus* specimens and the Brazilian crotalic reference venom | (59) |
| 2020 | Fusco et. al. | Fast venomic analysis of *Crotalus durissus terrificus* from north-eastern Argentina | (60) |
| 2018 | Damm et.al. | Comprehensive Snake Venomics of the Okinawa Habu pit Viper, Protobothrops flavoviridis, by complementary Mass- Spectrometry-Guided Approaches | (61) |
| 2018 | Mackessy et. al. | Venom ontogeny in the Mexican Lance-headed Rattlesnake (*Crotalus polystictus*). | (62) |
| 2019 | Chen et. al | Snake venom proteome and immuno-profiling of the hundred pace viper *Deinagkistrodon acutus*, in Taiwan | (63) |
| 2019 | Mendez et. al. | Proteomic profiling, functional characterization, and immunoneutralization of the venom of Porthidium porrasi, a pitviper endemic to Costa Rica | (64) |
| 2020 | Sanz et. al. | Venomics and antivenomics of the poorly studied Brazil’s lancehead, *Bothrops brazili* (Hoge, 1954), from the Brazilian State of Para | (65) |
| 2020 | Garcia-Osorio et. al. | Ontogenetic changes in the venom of *Metlapilcoatlus nummifer*, the Mexican Jumping Viper | (66) |
| 2018 | Amazonas et.al. | Molecular mechanisms underlying intraspecific variation in snake venom | (67) |
|  |  | **NON-FRONT-FANGED SNAKES** |  |
| 2018 | Modahl et. al. | Transcriptome-facilitated proteomic characterisation of rear-fanged snake venoms reveal abundant metalloproteases with enhanced activity. | (68) |
| 2020 | Calvete et. al. | Venomics of the Duvernoy’s gland secretion of the false coral snake *Rhinobothryum bovallii* (Anderson, 1916) and assessment of venom lethality towards synapsid and diapsid animal models. | (69) |
| 2020 | Mackessy et. al. | Venomics of the Central American Lyre Snake *Trimorphodon quadruplex* ( Colubridae: Smith, 1941) from Costa Rica. | (70) |
| 2018 | Pla et. al. | Transcriptomics-guided bottom-up and top-down venomics of neonate and adult specimens of the arboreal rear-fanged Brown Treesnake, Boiga irregularis, from Guam | (71) |
|  |  | **EXCLUSIVELY TOXIN DIVERSITY STUDIES** |  |
| 2018 | Kunalan et. al. | Proteomic characterisation of two medically important Malaysian snake venoms, *Calloselasma rhodostoma* (Malayan Pit Viper) and *Ophiophagus hannah* (King Cobra). | (72) |
| 2020 | Manuwar et. al. | Proteomic investigations of two Pakistani *Naja* snake venoms species unravel the venom complexity, post-translational modifications, and presence of extracellular vesicles. | (73) |
| 2018 | Olamendi-Portugal et. al. | New insights into the proteomic characterisation of the coral snake *Micrurus pyrrhocryptus* venom | (74) |
| 2020 | Katali et. al. | Protein identification of venoms of the African Spitting Cobras *Naja mossambica* and *Naja nigricincta nigricincta* | (75) |
| 2017 | Choudhury et. al. | Comparison of proteomic profiles of the venoms of two of the “Big Four” snakes of India, the Indian cobra (*Naja naja*) and the common krait (*Bungarus caeruleus*), and analyses of their toxins. | (76) |
| 2020 | Kumkate et. al. | Venomics and cellular toxicity of Thai Pit Vipers (*Trimeresurus macrops* and *T. hageni*). | (77) |
| 2021 | Arnaud et. al. | Proteomic comparison of adult and juvenile Santa Catalina rattlesnake (*Crotalus catalinensis*) venom | (78) |
| 2018 | Vanuopadath et. al. | Mass spectrometry-assisted venom profiling of Hypnale hypnale found in theWestern Ghats of India incorporating de- novo sequencing approaches | (79) |
| 2020 | Vanuopadath et. al. | Delineating the venom toxin arsenal of Malabar pit viper (Trimeresurus malabaricus) from the Western Ghats of India and evaluating its immunological cross-reactivity and in vitro cytotoxicity | (80) |
| 2021 | Gopcevich et.al. | Study of the venom proteome of *Vipera ammodytes ammodytes* (Linnaeus, 1758): A qualitative overview, biochemical and biological profiling | (81) |

1. Wang CR, Bubner ER, Jovcevski B, Mittal P, Pukala TL. Interrogating the higher order structures of snake venom proteins using an integrated mass spectrometric approach. Journal of Proteomics. 2020;216:103680.

2. Neale V, Sotillo J, Seymour JE, Wilson D. The Venom of the Spine-Bellied Sea Snake (Hydrophis curtus): Proteome, Toxin Diversity and Intraspecific Variation. International Journal of Molecular Sciences. 2017;18(12):2695.

3. Tan CH, Tan KY, Ng TS, Sim SM, Tan NH. Venom Proteome of Spine-Bellied Sea Snake (Hydrophis curtus) from Penang, Malaysia: Toxicity Correlation, Immunoprofiling and Cross-Neutralization by Sea Snake Antivenom. Toxins. 2019;11(1):3.

4. Wang B, Wang Q, Wang C, Wang B, Qiu L, Zou S, et al. A comparative analysis of the proteomes and biological activities of the venoms from two sea snakes, Hydrophis curtus and Hydrophis cyanocinctus, from Hainan, China. Toxicon. 2020;187:35-46.

5. Tan KY, Liew JL, Tan NH, Quah ESH, Ismail AK, Tan CH. Unlocking the secrets of banded coral snake (Calliophis intestinalis, Malaysia): A venom with proteome novelty, low toxicity and distinct antigenicity. Journal of Proteomics. 2019;192:246-57.

6. Patra A, Chanda A, Mukherjee AK. Quantitative proteomic analysis of venom from Southern India common krait (Bungarus caeruleus) and identification of poorly immunogenic toxins by immune-profiling against commercial antivenom. Expert Review of Proteomics. 2019;16(5):457-69.

7. Hia YL, Tan KY, Tan CH. Comparative venom proteomics of banded krait (Bungarus fasciatus) from five geographical locales: Correlation of venom lethality, immunoreactivity and antivenom neutralization. Acta Tropica. 2020;207:105460.

8. Chapeaurouge A, Silva A, Carvalho P, McCleary RJR, Modahl CM, Perales J, et al. Proteomic Deep Mining the Venom of the Red-Headed Krait, Bungarus flaviceps. Toxins. 2018;10(9):373.

9. Oh AMF, Tan CH, Tan KY, Quraishi NH, Tan NH. Venom proteome of Bungarus sindanus (Sind krait) from Pakistan and in vivo cross-neutralization of toxicity using an Indian polyvalent antivenom. Journal of Proteomics. 2019;193:243-54.

10. Sunagar K, Khochare S, Senji Laxme RR, Attarde S, Dam P, Suranse V, et al. A Wolf in Another Wolf’s Clothing: Post-Genomic Regulation Dictates Venom Profiles of Medically-Important Cryptic Kraits in India. Toxins. 2021;13(1):69.

11. Ainsworth S, Petras D, Engmark M, Süssmuth RD, Whiteley G, Albulescu L-O, et al. The medical threat of mamba envenoming in sub-Saharan Africa revealed by genus-wide analysis of venom composition, toxicity and antivenomics profiling of available antivenoms. Journal of Proteomics. 2018;172:173-89.

12. Tan KY, Wong KY, Tan NH, Tan CH. Quantitative proteomics of Naja annulifera (sub-Saharan snouted cobra) venom and neutralization activities of two antivenoms in Africa. International Journal of Biological Macromolecules. 2020;158:605-16.

13. Chanda A, Mukherjee AK. Quantitative proteomics to reveal the composition of Southern India spectacled cobra (Naja naja) venom and its immunological cross-reactivity towards commercial antivenom. International Journal of Biological Macromolecules. 2020;160:224-32.

14. Chanda A, Kalita B, Patra A, Senevirathne WDST, Mukherjee AK. Proteomic analysis and antivenomics study of Western India Naja naja venom: correlation between venom composition and clinical manifestations of cobra bite in this region. Expert Review of Proteomics. 2019;16(2):171-84.

15. Chanda A, Patra A, Kalita B, Mukherjee AK. Proteomics analysis to compare the venom composition between Naja naja and Naja kaouthia from the same geographical location of eastern India: Correlation with pathophysiology of envenomation and immunological cross-reactivity towards commercial polyantivenom. Expert Review of Proteomics. 2018;15(11):949-61.

16. Wong KY, Tan CH, Tan KY, Quraishi NH, Tan NH. Elucidating the biogeographical variation of the venom of Naja naja (spectacled cobra) from Pakistan through a venom-decomplexing proteomic study. Journal of Proteomics. 2018;175:156-73.

17. Tan CH, Wong KY, Chong HP, Tan NH, Tan KY. Proteomic insights into short neurotoxin-driven, highly neurotoxic venom of Philippine cobra (Naja philippinensis) and toxicity correlation of cobra envenomation in Asia. Journal of Proteomics. 2019;206:103418.

18. Wong KY, Tan KY, Tan NH, Tan CH. A Neurotoxic Snake Venom without Phospholipase A2: Proteomics and Cross-Neutralization of the Venom from Senegalese Cobra, Naja senegalensis (Subgenus: Uraeus). Toxins. 2021;13(1):60.

19. Adamude FA, Dingwoke EJ, Abubakar MS, Ibrahim S, Mohamed G, Klein A, et al. Proteomic analysis of three medically important Nigerian Naja (Naja haje, Naja katiensis and Naja nigricollis) snake venoms. Toxicon. 2021;197:24-32.

20. Sánchez A, Herrera M, Villalta M, Solano D, Segura Á, Lomonte B, et al. Proteomic and toxinological characterization of the venom of the South African Ringhals cobra Hemachatus haemachatus. Journal of Proteomics. 2018;181:104-17.

21. Whiteley G, Casewell NR, Pla D, Quesada-Bernat S, Logan RAE, Bolton FMS, et al. Defining the pathogenic threat of envenoming by South African shield-nosed and coral snakes (genus Aspidelaps), and revealing the likely efficacy of available antivenom. Journal of Proteomics. 2019;198:186-98.

22. Bénard-Valle M, Neri-Castro E, Yañez-Mendoza MF, Lomonte B, Olvera A, Zamudio F, et al. Functional, proteomic and transcriptomic characterization of the venom from Micrurus browni browni: Identification of the first lethal multimeric neurotoxin in coral snake venom. Journal of Proteomics. 2020;225:103863.

23. Sanz L, Quesada-Bernat S, Ramos T, Casais-e-Silva LL, Corrêa-Netto C, Silva-Haad JJ, et al. New insights into the phylogeographic distribution of the 3FTx/PLA2 venom dichotomy across genus Micrurus in South America. Journal of Proteomics. 2019;200:90-101.

24. Lippa E, Török F, Gómez A, Corrales G, Chacón D, Sasa M, et al. First look into the venom of Roatan Island's critically endangered coral snake Micrurus ruatanus: Proteomic characterization, toxicity, immunorecognition and neutralization by an antivenom. Journal of Proteomics. 2019;198:177-85.

25. Sanz L, de Freitas-Lima LN, Quesada-Bernat S, Graça-de-Souza VK, Soares AM, Calderón LdA, et al. Comparative venomics of Brazilian coral snakes: Micrurus frontalis, Micrurus spixii spixii, and Micrurus surinamensis. Toxicon. 2019;166:39-45.

26. Hus K, Marczak L, Petrilla V, Petrillova M, Legath J, Bocian A. Different Research approaches in Unravelling the Venom Proteome of naja ashei. Biomolecules. 2020;10(9):1282.

27. Senji Laxme RR, Attarde S, Khochare S, Suranse V, Martin G, Casewell NR, et al. Biogeographical venom variation in the Indian spectacled cobra (Naja naja) underscores the pressing need for pan-India efficacious snakebite therapy. PLoS Neglected Tropical Diseases. 2021;15(2):1-28.

28. Pla D, Quesada-Bernat S, Rodriguez Y, Sanchez A, Vargas M, Villata M, et al. Dagestan blunt-nosed viper, Macrovipera lebetina obtusa (Dwigubsky, 1832), venom. Venomics, antivenomics, and neutralisation assays of the lethal and toxic venom activities by anti- Macrovipera lebetina turanica and anti-Vipera berus berus antivenoms. Toxicon X. 2020;6.

29. Bhatia S, Vasudevan K. Comparative proteomics of geographically distinct saw-scaled viper (Echis carinatus) venoms from India. Toxicon X. 2020;7.

30. Patra A, Mukherjee AK. Proteomic Analysis of Sri Lanka Echis carinatus Venom: Immunological Cross-Reactivity and Enzyme Neutralization Potency of Indian Polyantivenom. Journal of Proteome Research. 2020;19(8):3022-32.

31. Ghezellou P, Albuquerque W, Garikapati V, Casewell NR, Kazemi SM, Ghassempour A, et al. Integrating Top-Down and Bottom-Up Mass Spectrometric Strategies for Proteomic Profiling of Iranian Saw-Scaled Viper, Echis carinatus sochureki, Venom. Journal of Proteome Research. 2021;20(1):895-908.

32. Ozverel CS, Damm M, Hempel B-F, Göçmen B, Sroka R, Süssmuth RD, et al. Investigating the cytotoxic effects of the venom proteome of two species of the Viperidae family (Cerastes cerastes and Cryptelytrops purpureomaculatus) from various habitats. Comparative Biochemistry and Physiology Part C: Toxicology & Pharmacology. 2019;220:20-30.

33. Pla D, Sanz L, Quesada-Bernat S, Villalta M, Baal J, Chowdhury MAW, et al. Phylovenomics of Daboia russelii across the Indian subcontinent. Bioactivities and comparative in vivo neutralization and in vitro third-generation antivenomics of antivenoms against venoms from India, Bangladesh and Sri Lanka. Journal of Proteomics. 2019;207:103443.

34. Faisal T, Tan KY, Sim SM, Quraishi N, Tan NH, Tan CH. Proteomics, functional characterization and antivenom neutralization of the venom of Pakistani Russell's viper (Daboia russelii) from the wild. Journal of Proteomics. 2018;183:1-13.

35. Kalita B, Patra A, Das A, Mukherjee AK. Proteomic Analysis and Immuno-Profiling of Eastern India Russell’s Viper (Daboia russelii) Venom: Correlation between RVV Composition and Clinical Manifestations Post RV Bite. Journal of Proteome Research. 2018;17(8):2819-33.

36. Sanz L, Quesada-Bernat S, Chen PY, Lee CD, Chiang JR, Calvete JJ. Translational Venomics: Third-Generation Antivenomics of Anti-Siamese Russell’s Viper, Daboia siamensis, Antivenom Manufactured in Taiwan CDC’s Vaccine Center. Tropical Medicine and Infectious Disease. 2018;3(2):66.

37. Hempel B-F, Damm M, Mrinalini, Göçmen B, Karış M, Nalbantsoy A, et al. Extended Snake Venomics by Top-Down In-Source Decay: Investigating the Newly Discovered Anatolian Meadow Viper Subspecies, Vipera anatolica senliki. Journal of Proteome Research. 2020;19(4):1731-49.

38. Giribaldi J, Kazandjian T, Amorim FG, Whiteley G, Wagstaff SC, Cazals G, et al. Venomics of the asp viper Vipera aspis aspis from France. Journal of Proteomics. 2020;218:103707.

39. Al-Shekhadat R, Lopushanskaya K, Segura A, Gutierrez J, Calvete J, Pla D. Vipera berus berus venom from Russia: Venomics, bioactivities and preclinical assessment of Microgen antivenom. Toxins. 2019;11 (2).

40. Hempel B-F, Damm M, Gocmen B, Karis M, Oguz MA, Nalbantsoy A, et al. Comparative venomics of the Vipera ammodytes trancaucasiana and Vipera ammodytes motandoni from Turkey provides insights into kinship. Toxins. 2018;10(1).

41. Petras D, Hempel B-F, Göçmen B, Karis M, Whiteley G, Wagstaff SC, et al. Intact protein mass spectrometry reveals intraspecies variations in venom composition of a local population of Vipera kaznakovi in Northeastern Turkey. Journal of Proteomics. 2019;199:31-50.

42. Lang Balija M, Leonardi A, Brgles M, Sviben D, Kurtović T, Halassy B, et al. Biological Activities and Proteomic Profile of the Venom of Vipera ursinii ssp., a very Rare Karst Viper from Croatia. Toxins. 2020;12(3):187.

43. Lingam TMC, Tan KY, Tan CH. Proteomics and antivenom immunoprofiling of Russell's Viper (Daboia siamensis) venoms from Thailand and Indonesia. Journal of Venomous Animals and Toxins including Tropical Diseases. 2020;26.

44. Senji Laxme RR, Khochare S, Attarde S, Suranse V, Iyer A, Casewell NR, et al. Biogeographic venom variation in Russell’s viper (Daboia russelii) and the preclinical inefficacy of antivenom therapy in snakebite hotspots. PLOS Neglected Tropical Diseases. 2021;15(3):e0009247.

45. Tan KY, Tan NH, Tan CH. Venom proteomics and antivenom neutralization for the Chinese eastern Russell’s viper, Daboia siamensis from Guangxi and Taiwan. Scientific Reports. 2018;8(1):8545.

46. Tang ELH, Tan NH, Fung SY, Tan CH. Comparative proteomes, immunoreactivities and neutralization of procoagulant activities of Calloselasma rhodostoma (Malayan pit viper) venoms from four regions in Southeast Asia. Toxicon. 2019;169:91-102.

47. Jones BK, Saviola AJ, Reilly SB, Stubbs AL, Arida E, Iskandar DT, et al. Venom Composition in a Phenotypically Variable Pit Viper (Trimeresurus insularis) across the Lesser Sunda Archipelago. Journal of Proteome Research. 2019;18(5):2206-20.

48. Tan CH, Tan KY, Ng TS, Quah ESH, Ismail AK, Khomvilai S, et al. Venomics of Trimeresurus (Popeia) nebularis, the Cameron Highlands Pit viper from Malayasia: Insights into venom proteome, toxicity, and neutralisation of antivenom. Toxins. 2019;11(2).

49. Liu C-C, Wu C-J, Hsiao Y-C, Yang Y-H, Liu K-L, Huang G-J, et al. Snake venom proteome of Protobothrops mucrosquamatus in Taiwan: Delaying venom-induced lethality in a rodent model by inhibition of phospholipase A2 activity with varespladib. Journal of Proteomics. 2021;234:104084.

50. Mora-Obando D, Salazar-Valenzuela D, Pla D, Lomonte B, Guerrero-Vargas JA, Ayerbe S, et al. Venom variation in Bothrops asper lineages from North-Western South America. Journal of Proteomics. 2020;229:103945.

51. Sanz L, Quesada-Bernat S, Pérez A, De Morais-Zani K, SantˈAnna SS, Hatakeyama DM, et al. Danger in the Canopy. Comparative Proteomics and Bioactivities of the Venoms of the South American Palm Pit Viper Bothrops bilineatus Subspecies bilineatus and smaragdinus and Antivenomics of B. b. bilineatus (Rondônia) Venom against the Brazilian Pentabothropic Antivenom. Journal of Proteome Research. 2020;19(8):3518-32.

52. Rodrigues CR, Teixeira-Ferreira A, Vargas FFR, Guerra-Duarte C, Costal-Oliveira F, Stransky S, et al. Proteomic profile, biological activities and antigenic analysis of the venom from Bothriopsis bilineata smaragdina (“loro machaco”), a pitviper snake from Peru. Journal of Proteomics. 2018;187:171-81.

53. Rodrigues CR, Molina DAM, Silva de Assis TC, Liberato C, Melo-Braga MN, Ferreyra CB, et al. Proteomic and toxinological characterization of Peruvian pitviper Bothrops brazili ("jergón shushupe"), venom. Toxicon. 2020;184:19-27.

54. Galizio NdC, Serino-Silva C, Stuginski DR, Abreu PAE, Sant'Anna SS, Grego KF, et al. Compositional and functional investigation of individual and pooled venoms from long-term captive and recently wild-caught Bothrops jararaca snakes. Journal of Proteomics. 2018;186:56-70.

55. Pereañez JA, Preciado LM, Fernández J, Camacho E, Lomonte B, Castro F, et al. Snake venomics, experimental toxic activities and clinical characteristics of human envenomation by Bothrocophias myersi (Serpentes: Viperidae) from Colombia. Journal of Proteomics. 2020;220:103758.

56. Neri-Castro E, Sanz L, Olvera-Rodríguez A, Bénard-Valle M, Alagón A, Calvete JJ. Venomics and biochemical analysis of the black-tailed horned pitviper, Mixcoatlus melanurus, and characterization of Melanurutoxin, a novel crotoxin homolog. Journal of Proteomics. 2020;225:103865.

57. Neri-Castro E, Lomonte B, Valdés M, Ponce-López R, Bénard-Valle M, Borja M, et al. Venom characterization of the three species of Ophryacus and proteomic profiling of O. sphenophrys unveils Sphenotoxin, a novel Crotoxin-like heterodimeric β-neurotoxin. Journal of Proteomics. 2019;192:196-207.

58. Quintana-Castillo JC, Vargas LJ, Segura C, Estrada-Gómez S, Bueno-Sánchez JC, Alarcón JC. Characterization of the Venom of C. d. cumanesis of Colombia: Proteomic Analysis and Antivenomic Study. Toxins. 2018;10(2):85.

59. Tasima LJ, Hatakeyama DM, Serino-Silva C, Rodrigues CFB, de Lima EOV, Sant'Anna SS, et al. Comparative proteomic profiling and functional characterization of venom pooled from captive Crotalus durissus terrificus specimens and the Brazilian crotalic reference venom. Toxicon. 2020;185:26-35.

60. Fusco LS, Neto EB, Francisco AF, Alfonso J, Soares A, Pimenta DC, et al. Fast venomic analysis of Crotalus durissus terrificus from northeastern Argentina. Toxicon: X. 2020;7:100047.

61. Damm M, Hempel B-F, Nalbantsoy A, Süssmuth RD. Comprehensive Snake Venomics of the Okinawa Habu Pit Viper, Protobothrops flavoviridis, by Complementary Mass Spectrometry-Guided Approaches. Molecules. 2018;23(8):1893.

62. Mackessy SP, Leroy J, Mociño-Deloya E, Setser K, Bryson RW, Saviola AJ. Venom Ontogeny in the Mexican Lance-Headed Rattlesnake (Crotalus polystictus). Toxins (Basel). 2018;10(7).

63. Chen P-C, Huang M-N, Chang J-F, Liu C-C, Chen C-K, Hsieh C-H. Snake venom proteome and immuno-profiling of the hundred-pace viper, Deinagkistrodon acutus, in Taiwan. Acta Tropica. 2019;189:137-44.

64. Méndez R, Bonilla F, Sasa M, Dwyer Q, Fernández J, Lomonte B. Proteomic profiling, functional characterization, and immunoneutralization of the venom of Porthidium porrasi, a pitviper endemic to Costa Rica. Acta Tropica. 2019;193:113-23.

65. Sanz L, Perez A, Quesada-Bernat S, Diniz-Sousa R, Calderon LA, Soares AM, et al. Venomics and Antivenomics of the poorly studied Brazil's Lancehead *Bothrops brazili* (Hoge, 1954), from the Brazilian state of Para. Journal of Venomous Animals and Toxins including Tropical Diseases. 2020;26.

66. García-Osorio B, Lomonte B, Bénard-Valle M, López de León J, Román-Domínguez L, Mejía-Domínguez NR, et al. Ontogenetic changes in the venom of Metlapilcoatlus nummifer, the mexican jumping viper. Toxicon. 2020;184:204-14.

67. Amazonas DR, Portes-Junior JA, Nishiyama-Jr MY, Nicolau CA, Chalkidis HM, Mourão RHV, et al. Molecular mechanisms underlying intraspecific variation in snake venom. J Proteomics. 2018;181:60-72.

68. Modahl CM, Frietze S, Mackessy SP. Transcriptome-facilitated proteomic characterization of rear-fanged snake venoms reveal abundant metalloproteinases with enhanced activity. Journal of Proteomics. 2018;187:223-34.

69. Calvete JJ, Bonilla F, Granados-Martínez S, Sanz L, Lomonte B, Sasa M. Venomics of the Duvernoy's gland secretion of the false coral snake Rhinobothryum bovallii (Andersson, 1916) and assessment of venom lethality towards synapsid and diapsid animal models. Journal of Proteomics. 2020;225:103882.

70. Mackessy SP, Bryan W, Smith CF, Lopez K, Fernández J, Bonilla F, et al. Venomics of the Central American Lyre Snake Trimorphodon quadruplex (Colubridae: Smith, 1941) from Costa Rica. Journal of Proteomics. 2020;220:103778.

71. Pla D, Petras D, Saviola AJ, Modahl CM, Sanz L, Pérez A, et al. Transcriptomics-guided bottom-up and top-down venomics of neonate and adult specimens of the arboreal rear-fanged Brown Treesnake, Boiga irregularis, from Guam. Journal of Proteomics. 2018;174:71-84.

72. Kunalan S, Othman I, Syed Hassan S, Hodgson WC. Proteomic Characterization of Two Medically Important Malaysian Snake Venoms, Calloselasma rhodostoma (Malayan Pit Viper) and Ophiophagus hannah (King Cobra). Toxins. 2018;10(11):434.

73. Manuwar A, Dreyer B, Böhmert A, Ullah A, Mughal Z, Akrem A, et al. Proteomic Investigations of Two Pakistani Naja Snake Venoms Species Unravel the Venom Complexity, Posttranslational Modifications, and Presence of Extracellular Vesicles. Toxins. 2020;12(11):669.

74. Olamendi-Portugal T, Batista CVF, Pedraza-Escalona M, Restano-Cassulini R, Zamudio FZ, Benard-Valle M, et al. New insights into the proteomic characterization of the coral snake Micrurus pyrrhocryptus venom. Toxicon. 2018;153:23-31.

75. Katali O, Shipingana L, Nyarangó P, Pääkkönen M, Haindongo E, Rennie T, et al. Protein Identification of Venoms of the African Spitting Cobras, Naja mossambica and Naja nigricincta nigricincta. Toxins. 2020;12(8):520.

76. Choudhury M, McCleary RJR, Kesherwani M, Kini RM, Velmurugan D. Comparison of proteomic profiles of the venoms of two of the ‘Big Four’ snakes of India, the Indian cobra (Naja naja) and the common krait (Bungarus caeruleus), and analyses of their toxins. Toxicon. 2017;135:33-42.

77. Kumkate S, Chanhome L, Thiangtrongjit T, Noiphrom J, Laoungboa P, Khow O, et al. Venomics and Cellular Toxicity of Thai Pit Vipers (Trimeresurus macrops and T. hageni). Toxins (Basel). 2020;12(1).

78. Arnaud G, García-de León FJ, Beltrán LF, Carbajal-Saucedo A. Proteomic comparison of adult and juvenile Santa Catalina rattlesnake (Crotalus catalinensis) venom. Toxicon. 2021;193:55-62.

79. Vanuopadath M, Sajeev N, Murali AR, Sudish N, Kangosseri N, Sebastian IR, et al. Mass spectrometry-assisted venom profiling of Hypnale hypnale found in the Western Ghats of India incorporating de novo sequencing approaches. International Journal of Biological Macromolecules. 2018;118:1736-46.

80. Vanuopadath M, Shaji SK, Raveendran D, Nair BG, Nair SS. Delineating the venom toxin arsenal of Malabar pit viper (Trimeresurus malabaricus) from the Western Ghats of India and evaluating its immunological cross-reactivity and in vitro cytotoxicity. International Journal of Biological Macromolecules. 2020;148:1029-45.

81. Gopcevic K, Karadzic I, Izrael-Zivkovic L, Medic A, Isakovic A, Popović M, et al. Study of the venom proteome of Vipera ammodytes ammodytes (Linnaeus, 1758): A qualitative overview, biochemical and biological profiling. Comparative Biochemistry and Physiology Part D: Genomics and Proteomics. 2021;37:100776.
